# Effects of different habitual foot strike patterns on *in vivo* kinematics of the first metatarsophalangeal joint during shod running—a statistical parametric mapping study

**DOI:** 10.3389/fbioe.2023.1251324

**Published:** 2023-09-06

**Authors:** Kaicheng Wu, Xiaole Sun, Dongqiang Ye, Faning Zhang, Shen Zhang, Weijie Fu

**Affiliations:** ^1^ School of Exercise and Health, Shanghai University of Sport, Shanghai, China; ^2^ School of Sports and Health, Nanjing Sport Institute, Nanjing, China; ^3^ Shanghai Warrior Shoes Co., Ltd., Shanghai, China; ^4^ School of Athletic Performance, Shanghai University of Sport, Shanghai, China; ^5^ Key Laboratory of Exercise and Health Sciences of Ministry of Education, Shanghai University of Sport, Shanghai, China

**Keywords:** rearfoot strike, forefoot strike, shod running, 1st metatarsophalangeal joint, *in vivo* kinematics

## Abstract

Existing studies on the biomechanical characteristics of the first metatarsophalangeal joint (1st MTPJ) during shod running are limited to sagittal plane assessment and rely on skin marker motion capture, which can be affected by shoes wrapping around the 1st MTPJ and may lead to inaccurate results. This study aims to investigate the *in vivo* effects of different habitual foot strike patterns (FSP) on the six degrees of freedom (6DOF) values of the 1st MTPJ under shod condition by utilizing a dual-fluoroscopic imaging system (DFIS). Long-distance male runners with habitual forefoot strike (FFS group, *n* = 15) and rearfoot strike (RFS group, *n* = 15) patterns were recruited. All participants underwent foot computed tomography (CT) scan to generate 3D models of their foot. The 6DOF kinematics of the 1st MTPJ were collected using a DFIS at 100 Hz when participants performed their habitual FSP under shod conditions. Independent t-tests and one-dimensional statistical parametric mapping (1-d SPM) were employed to analyze the differences between the FFS and RFS groups’ 1st MTPJ 6DOF kinematic values during the stance phase. FFS exhibited greater superior translation (3.5–4.9 mm, *p* = 0.07) during 51%–82% of the stance and higher extension angle (8.4°–10.1°, *p* = 0.031) during 65%–75% of the stance in the 1st MTPJ than RFS. Meanwhile, FFS exhibited greater maximum superior translation (+3.2 mm, *p* = 0.022), maximum valgus angle (+6.1°, *p =* 0.048) and varus–valgus range of motion (ROM) (+6.5°, *p =* 0.005) in the 1st MTPJ during stance. The greater extension angle of the 1st MTPJ in the late stance suggested that running with FFS may enhance the propulsive effect. However, the higher maximum valgus angle and the ROM of varus–valgus in FFS may potentially lead to the development of hallux valgus.

## 1 Introduction

The first metatarsophalangeal joint (1st MTPJ), referred to as the terminal joint of the foot, is a crucial contributor to running, particularly during the propulsion phase (Goldmann et al., 2011; Cigoja et al., 2022; Smith et al., 2022). It is an ellipsoidal synovial joint that connects the metatarsal bones to the proximal phalanges, permitting various movements, such as flexion, extension, varus, valgus, circumduction (pronation and supination) and translation (Stephen et al., 2010). During the gait cycle, the movement of the 1st MTPJ plays a vital role in propulsion. On the one hand, the extension of the 1st MTPJ can stretch the plantar fascia and lift the arch. This, in turn, increases the stiffness of the arch through a windlass mechanism, thereby aiding in the transmission of force to the ground (Hicks, 1954; Welte et al., 2021). On the other hand, the transverse plane movement of the 1st MTPJ influences the alignment and stability of the foot, ensuring the proper distribution of plantpressure and aiding in maintaining the balance during walking or running (Menz and Lord, 2005; Koller et al., 2014; Xiang et al., 2022). The rotational motion of the 1st MTPJ in sagittal plane has received significant research attention (Bruening et al., 2018; Chen et al., 2019). However, during the stance phase of running, the 1st MTPJ not only undergoes flexion and extension motion but also experiences passive movements of circumduction and varus and valgus motion (Shereff et al., 1986; Zhang et al., 2022).

Foot strike pattern (FSP) can alter the movement characteristics of the 1st MTPJ during running, thereby influencing the windlass mechanism. By utilizing a marker-based motion capture system, Bruening et al. observed that the 1st MTPJ transitioned into extension earlier during the late stance in forefoot strike pattern (FFS) compared to the rearfoot strike pattern (RFS) (Bruening et al., 2018). Furthermore, the angle of the MTPJ extension was greater in the FFS than in the RFS. The increased excursion of the MTPJ during late stance has the potential to enhance the structural rigidity of the medial longitudinal arch and improve the push-off. However, the presence of relative motion between the outer skin and underlying bone causes inaccuracies when using marker-based methods (Reinschmidt et al., 1997; Shultz et al., 2011). Additionally, there is also relative movement between the foot and the shoe under shod conditions, which further contributes to potential inaccuracies in the outcomes (Roach et al., 2021). Furthermore, the 1st MTPJ is relatively smaller when compared to the hip, knee and ankle joints. Therefore it may require higher precision in motion capture during *in vivo* kinematic analysis.

Recently, the dual-fluoroscopic imaging system (DFIS) has gradually been applied in the fields of sports analysis and injury prevention (Cao et al., 2019; Zhang et al., 2022; Ye et al., 2023). DFIS has the advantages of non-invasiveness, highly accurate testing, high repeatability and ability to dynamically capture skeletal *in vivo* movement; it is not affected by skin and soft tissue movement. The accuracy of DFIS in assessing joint translation and rotation reaches sub-millimetre (<0.1 mm) and sub-degree (<0.1°) levels, respectively (Cross et al., 2017), which break through the limitations of current traditional measurement methods in imaging technology and measurement accuracy.

This study aims to investigate the *in vivo* effects of different habitual FSP on the six degrees of freedom (6DOF) kinematic data of the 1st MTPJ under shod condition by utilizing DFIS. We hypothesized that habitual FFS runners will exhibit a greater extension angle and superior displacement in push-off phase compared with habitual RFS runners.

## 2 Materials and methods

### 2.1 Participants

**FIGURE 1:**
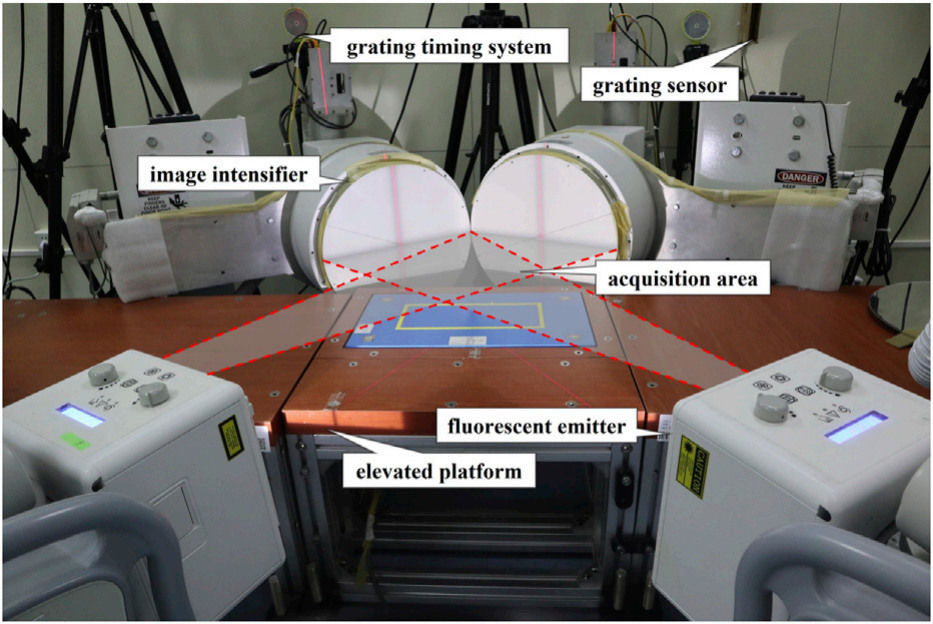
The setup of DFIS.

Thirty recreational long-distance male runners were recruited in this study, including 15 habitual FFS runners (age: 32.6 ± 8.5 years, height: 171.8 ± 4.7 cm, body mass: 65.0 ± 8.0 kg) and 15 comparable habitual RFS runners (age: 31.8 ± 6.8 years, height: 173.2 ± 4.3 cm, body mass: 71.6 ± 6.8 kg). FSP was determined using DFIS while the participants were running on an elevated platform (Figure 1). A posthoc power analysis was conducted in G*Power (v3.1.9.6, Univ. Kiel, Kiel, Schleswig-Holstein, Germany) and indicated that *n* = 15 (per group) would provide a statistical power of up to 90% with the sample size of the ROM of the 1st MTPJ for pronation and supination (effect size d = 1.104) and a type I error probability of 0.05. The following criteria were used for participant selection: i) habitual distance runners with RFS and FFS; ii) weekly running distance of at least 20 km; iii) no lower limb injuries or neurological disorders over the previous 6 months; iv) no vigorous activities within 24 h prior to the test. This study obtained ethical approval from the institutional review board of Shanghai University of Sport (No. 102772021RT034). The informed consent was obtained from all participants prior to the commencement of the official experiment.

### 2.2 Instrumentation

#### 2.2.1 CT

The participants’ feet were scanned using a 64-row 128-layer spiral CT scanner (Siemens AS+ 128, Somatom, Berlin, Germany) in the neutral position. The CT scan was performed with a slice thickness and interval of 0.6 mm. The voltage applied during the scan was 120 kV, and the current was set at 140 mA. The voxel size was configured to be 0.488 mm in length and width, and 0.625 mm in height. The resulting images had a resolution of 512 × 512 × 256 dots per inch (dpi).

#### 2.2.2 DFIS

The DFIS utilized in this study comprised two sets of fluoroscopic imaging systems (Figure 1). Each set consisted of an X-ray source responsible for generating X-rays, and a scintillator screen to receive and enhance the X-ray images. Two digital cameras (Phantom V5.1, Vision Research, New Jersey, United States) were equipped with built-in synchronization control devices, ensuring the synchronization of the imaging process. These cameras were configured to consistently operate with a shutter speed of 1/1,000 s. A specific scintillator screen with a diameter of 431.8 mm was selected. The spacing between the initial X-ray source and the scintillator screen was established at 132.2 cm, while the second X-ray source was positioned at a distance of 128.6 cm from the scintillator screen. Additionally, an angle of 120° was selected between the scintillator screens. The imaging parameters were configured as follows: the X-ray voltage used was 60 kV, with a current of 63 mA. The system operated at a capture frequency of 100 Hz. The resulting image resolution was 1,024 × 1,024 dpi.

### 2.3 Procedures

#### 2.3.1 CT scans of the foot

During the CT scan, the participant was in a supine posture, and a rigid immobilization device was adopted to secure the right foot with the shank perpendicular and the ankle angle at 90° (Zhang et al., 2022). The scanning range encompassed a region starting above the ankle joint and extending to the bottom of the calcaneus (Sun et al., 2022; Zhang et al., 2022). The acquired images were saved in DICOM format for subsequent three-dimensional (3D) model reconstruction of the first metatarsal and first phalangeal bones.

#### 2.3.2 Calibration of DFIS

The alignment of the centers of the scintillator screen and the X-ray source was adjusted. Images of a calibration cube and a grid plate were acquired, ensuring that at least 80% of the steel ball calibration points within the cube and four specific calibration points were captured. XMAlab software (v 1.5.4, Brown, United States) was employed to calculate the spatial relationship between the X-ray source and the image receiver.

#### 2.3.3 Running tests

The participants were instructed to change into shorts and traditional running shoes (6 mm heel-to-toe drop; TPU and EVA midsole; textile fabric upper; no arch support) and underwent a 5-min running session on a treadmill with a constant speed of 3 m/s. After that, the participants were instructed to complete the running task by using their habitual FSP. The participants ran at a speed of 2.85–3.15 m/s (3 m/s ± 5%) on the elevated platform while under the supervision of the experimenter to ensure they maintained straight eye-gaze and that their right foot landed in the acquisition area. If the landing position did not meet the requirements, the experimenter made adjustments to the participant’s starting line until they successfully completed at least two consecutive runs, thereby avoiding “targeting”. During the testing, the blocking grating sensor was obstructed by the participants to initiate the data collection process. Subsequently, X-ray images of the foot bones were captured by DFIS during the participants’ stance phase. The collected images were used to assess if the dominant foot of the participant was within the acquisition area (Zhang et al., 2022). A whole image of the foot during the stance phase was regarded as valid data if the right foot entered the acquisition area and the entire stance phase was captured. One valid data set was selected for each trial for analysis (Campbell et al., 2016; Welte et al., 2021; Zhang et al., 2022; Ye et al., 2023).

### 2.4 Data processing

#### 2.4.1 Creation of 3D bone models

**FIGURE 2:**
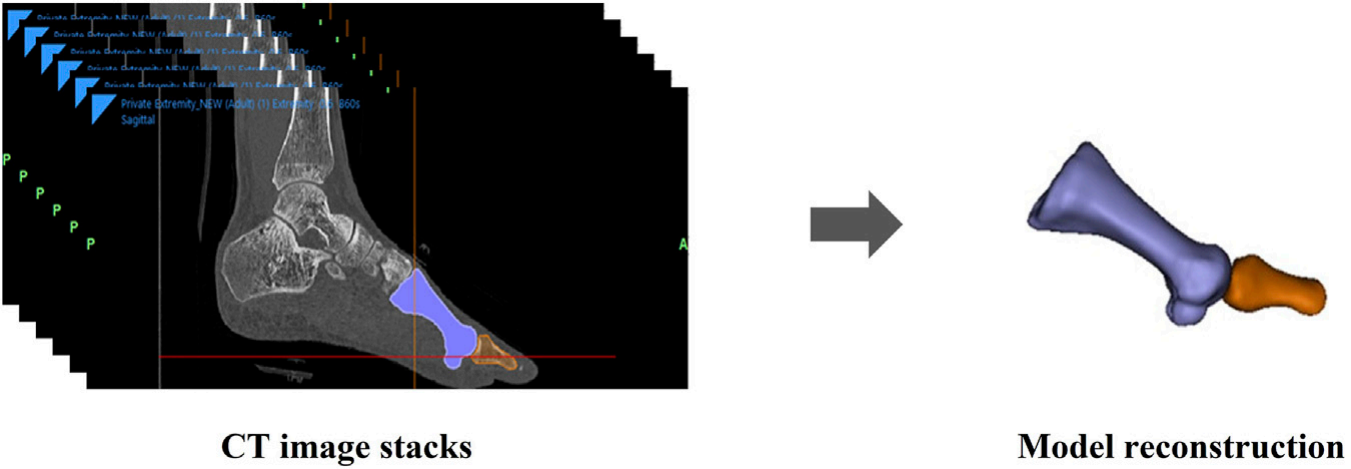
1st MTPJ reconstruction.

The foot CT scan images were processed using Mimics software (version: 21.0, Materialise, Belgium). Subsequently, by reconstructing the CT images, the operator used Mimics software to generate 3D bone models of the 1st MTPJ (first phalanx and first metatarsal). The joint surface underwent smoothing and noise reduction techniques, with an iteration of 2 and a smoothing factor of 0.4 (Figure 2).

#### 2.4.2 Coordinate system

Based on the anatomical inertial coordinate system, a local coordinate system for the first metatarsal bone and the first proximal phalanx was established (Sun et al., 2022; Zhang et al., 2022). The origin of the coordinate system was set at the centre of the bone mass, while the three coordinate axes were in accordance the principal axes of the bone’s inertia tensor (Zhang et al., 2022). Within this coordinate system, the X-axis corresponded to the direction of medial-lateral; the Y-axis indicated the direction of anterior-posterior; and the Z-axis represented the direction of superior-inferior.

#### 2.4.3 3D–2D registration

**FIGURE 3:**
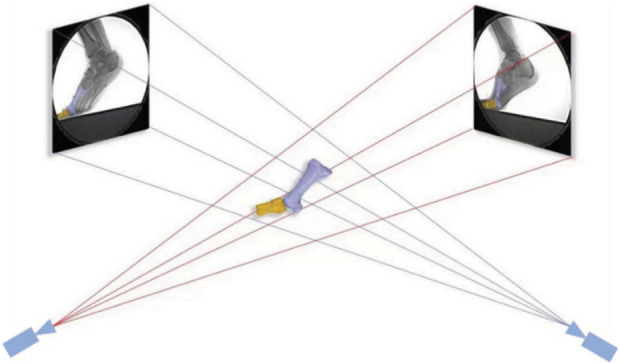
3D–2D registration.

The environment calibration file generated by XMAlab was loaded into the modeling Rhinoceros software (Rhinoceros: 6.0, Robert, United States). The shooting space was reconstructed in the virtual space by the modeling module, and the relative positions of the two sets of X-ray sources and the scintillator screen were restored. The images of the grid plate were captured and subsequently utilized by a circular aluminum plate featuring 406 perforations, securely affixed to the input side of the scintillator screen. This arrangement was employed to address pincushion distortion and magnetic lens distortion. Following this, the imaged locations of these perforations underwent processing via a thin plate spline algorithm subsequent to imaging with two X-ray sources (Fantozzi et al., 2003). After distortion correction, Adobe Photoshop CC 2018 (Adobe Systems, San Jose, CA, United States) was utilized for image enhancement, aiming to enhance the clarity of the bone outline. After that, the 3D bone models of the first metatarsal as well as the first proximal phalanx were generated, and the corresponding distortion-corrected fluorescent images of the foot were obtained. The bone models were adjusted through translation and rotation to achieve alignment with the bone contour observed in the fluorescent images (Cao et al., 2019) (Figure 3). Both our prior study and Cross et al.’s demonstrated a strong correlation (r > 0.917) between 6DOF joint kinematics obtained through DFIS and the gold standard results from bone pins (Cross et al., 2017; Zhang, 2020).

#### 2.4.4 Variables

**FIGURE 4:**
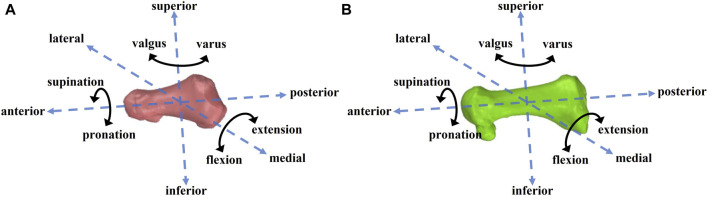
First proximal phalanx **(A)** and the first metatarsal **(B)** 6DOF motion diagram.

The 6DOF data of the 1st MTPJ were calculated using a plugin in Rhinoceros software. The data included information in three translational directions (medial–lateral, anterior–posterior, superior–inferior) and three rotational directions (extension–flexion, varus–valgus, pronation–supination) (Figure 4). The parameters included the 6DOF motion of the 1st MTPJ, the maximum and minimum translation, the maximum displacement (difference between maximum and minimum translation), the maximum rotation angles and minimum rotation angles, and the joint ROM (difference between maximum and minimum angles). Positive values indicated lateral translation, anterior translation, superior translation, extension, varus, and pronation of the positioning of the first proximal phalanx in relation to the first metatarsal. Conversely, negative values denoted the opposite direction of motion for each parameter. The entire 6DOF data of the 1st MTPJ during the stance phase were subjected to time normalization and filtered using MATLAB software (R2020a) with a cut-off frequency of 20 Hz (Welte et al., 2021).

### 2.5 Statistical analysis

The normal distribution was assessed using the Shapiro-Wilk test. The kinematic characteristics of the 6DOF motion of the 1st MTPJ were presented as mean ± standard deviation. One-dimensional statistical parametric mapping (1-d SPM) was employed to conduct a two-tailed, two-sample *t*-test (α = 0.05) for the 6DOF data of the 1st MTPJ between habitual FFS and habitual RFS (Yu et al., 2021; Deschamps et al., 2022). The outputs of SPM included a time series of t values, allowing to analyse differences across the whole stance phase (Pataky et al., 2015; Pataky et al., 2016). Independent t-tests were employed to analyze the differences in peak biomechanical variables across different FSP (α = 0.05).

## 3 Results

### 3.1 Translational movement of the 1st MTPJ

**FIGURE 5:**
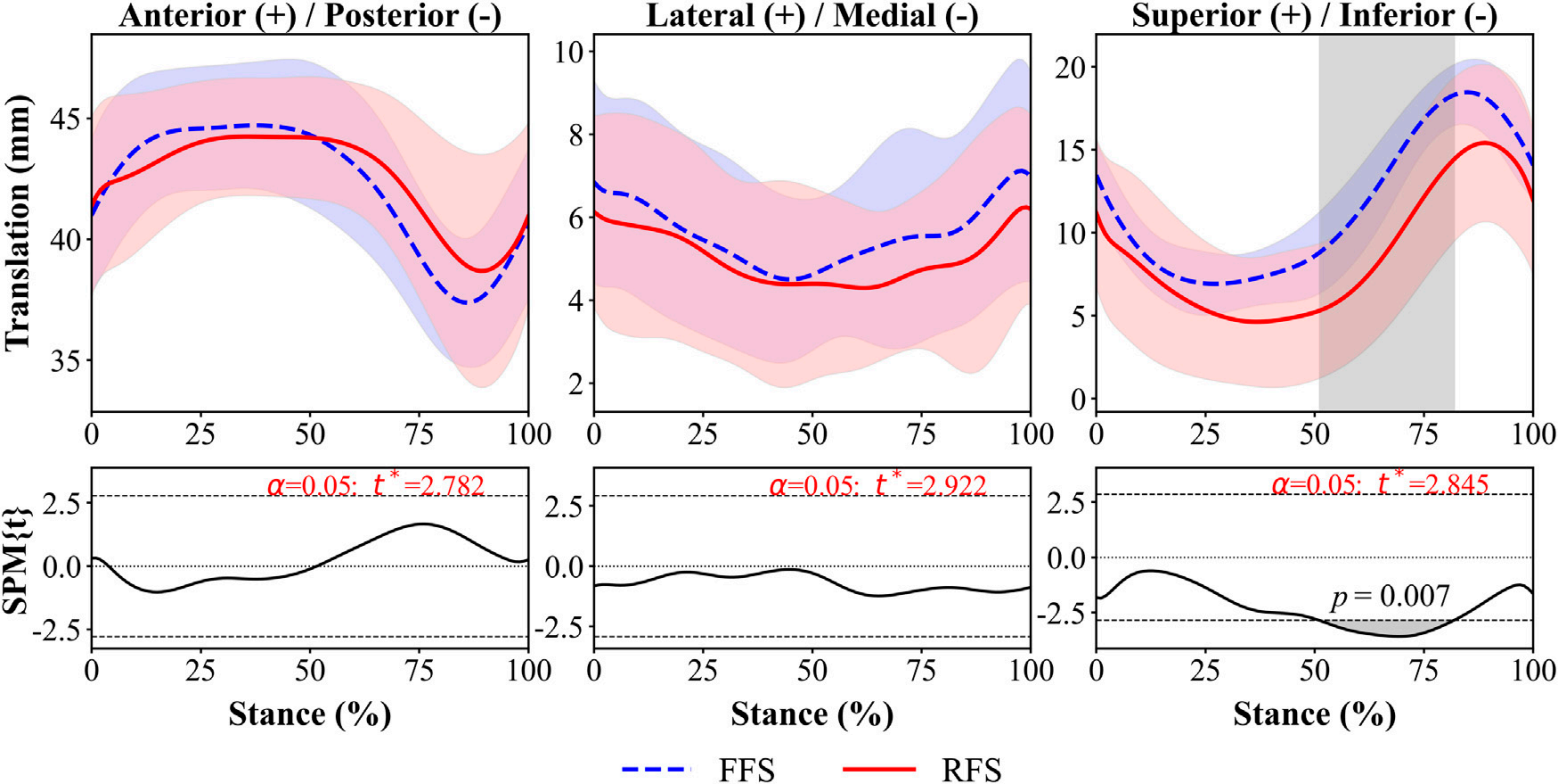
1st MTPJ *in vivo* kinematics for translation during running stance between FFS and RFS. Note: FFS, forefoot strike; RFS, rearfoot strike; the grey shaded areas represents the significant difference between two strike patterns, and the top and bottom black dashed line on the SPM figure represents *p =* 0.05.

Regardless of FSP, during the early stance phase, the 1st MTPJ moved medially, anteriorly and inferiorly and reached its maximum value during the mid-stance phase; it then began to move laterally, posteriorly and superiorly (Figure 5).

**FIGURE 6:**
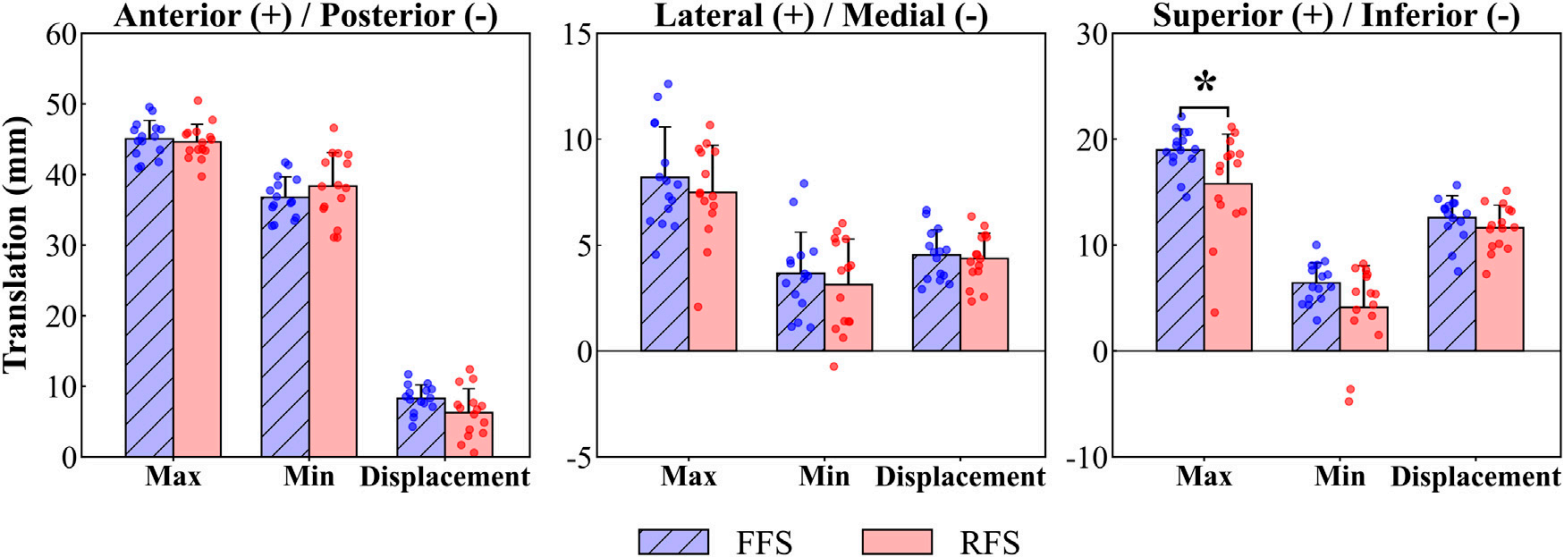
1st MTPJ translation characteristic values during running stance between FFS and RFS. Note: FFS, forefoot strike; RFS, rearfoot strike; Max, maximum; Min, minimum; Displacement, maximum displacement; *significant difference, *p* < 0.05.

The 1st MTPJ showed significantly greater superior translation (3.5–4.9 mm, 51%–82% of stance phase, *p =* 0.07, Figure 5) and maximum superior translation (3.2 mm, *p =* 0.022, Figure 6) in FFS than in RFS. No statistical significance was detected in other translational DOF of the 1st MTPJ.

### 3.2 Rotational movement of the 1st MTPJ

Regardless of FSP, during the early stance phase, the 1st MTPJ flexed and rotated in the varus direction and reached its maximum value during the mid-stance phase; it then began extending and rotating in the valgus direction.

**FIGURE 7:**
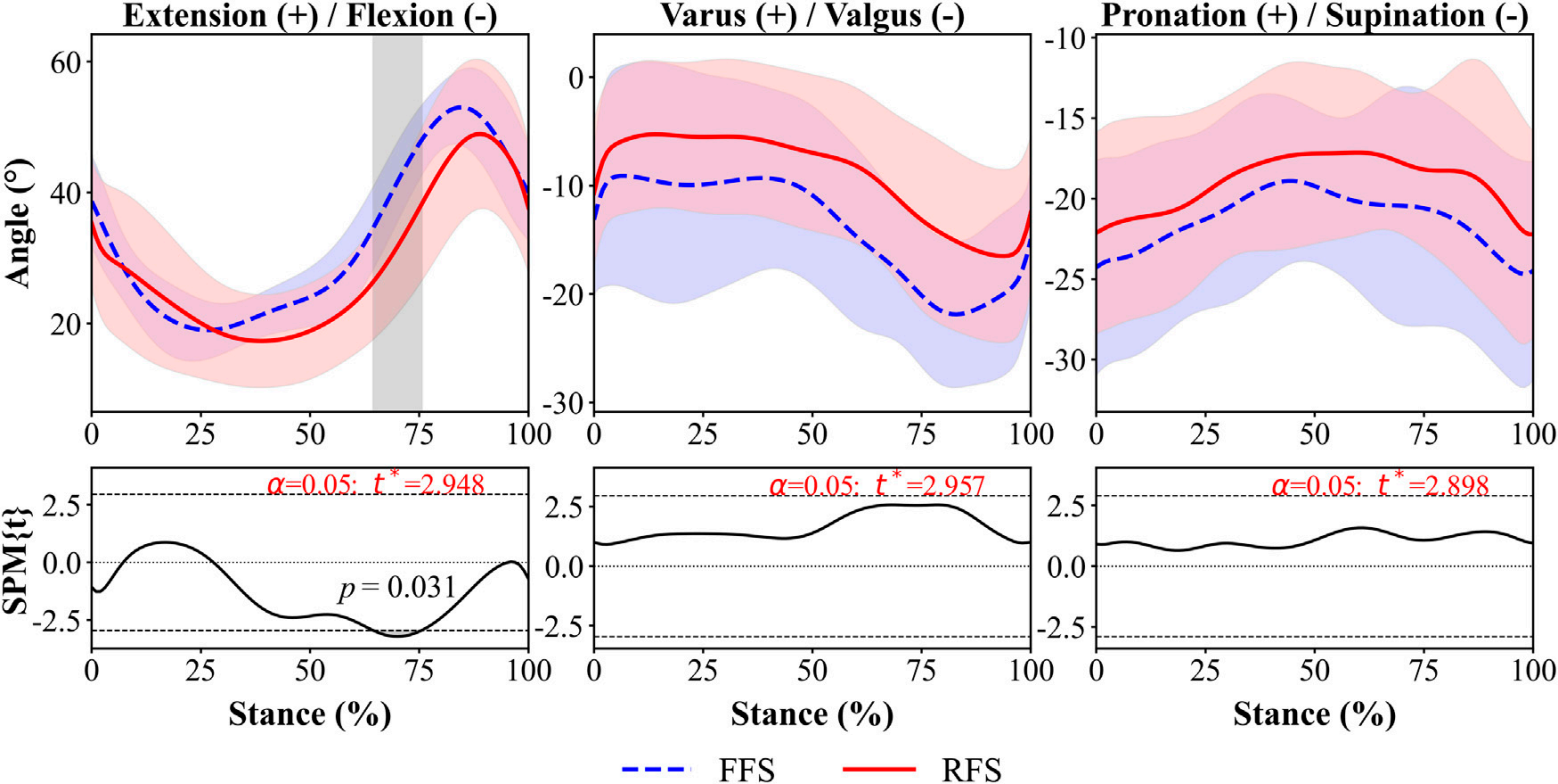
1st MTPJ *in vivo* kinematics for rotation during running stance between FFS and RFS. Note: FFS, forefoot strike; RFS, rearfoot strike; the grey shaded areas represents the significant difference between two strike patterns, and the top and bottom black dashed line on the SPM figure represents *p =* 0.05.

**FIGURE 8:**
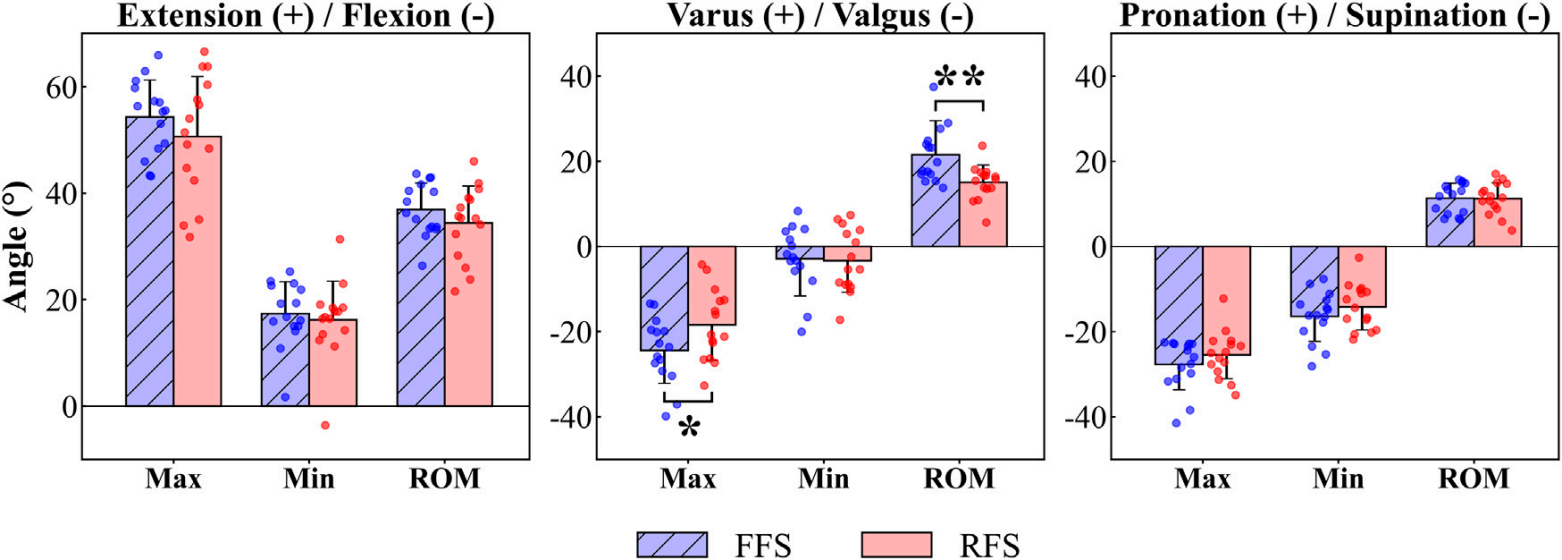
1st MTPJ rotation characteristic values during running stance between FFS and RFS. Note: FFS, forefoot strike; RFS, rearfoot strike; Max, maximum; Min, minimum; ROM, range of motion; *significant difference, *p* < 0.05.

The FFS group had a significantly greater extension angle (8.4°–10.1°, 65%–75% of stance phase, *p =* 0.031, Figure 7) of the 1st MTPJ, maximum valgus angle (6.1°, *p =* 0.048, Figure 8) and highest ROM from varus–valgus (6.5°, *p =* 0.005, Figure 8) than the RFS group. No statistical significance was detected in the other rotational DOF of the 1st MTPJ.

## 4 Discussion

To the best of our knowledge, this is the first study to utilize DFIS to investigate differences in the 6DOF kinematics of the 1st MTPJ between habitual runners with RFS and FFS patterns. During 51%–82% of the stance phase, the 1st MTPJ translation in FFS was significantly greater in the superior direction (3.5–4.9 mm) compared to that in RFS. The maximum superior translation in FFS was significantly larger than that in RFS. The extension of the 1st MTPJ in FFS showed a significantly higher magnitude (8.4°–10.1°) during 65%–75% of the stance phase, and the maximum valgus also varus–valgus ROM were significantly higher in FFS than in RFS. These outcomes partly align with our initial hypothesis.

In this study, during the stance phase of 0%–40% and 85%–100%, the flexion of the 1st MTPJ occurred while moving inferior; during the stance phase of 40%–85%, the extension of the 1st MTPJ occurred while moving superior. These findings provide support for previous research that the inferior translation of the first metatarsal maximises the extension of the 1st MTPJ during the stance phase (Michaud, 1993). In addition, the increased translation of the 1st MTPJ in superior direction during the 40%–85% of the stance phase was observed in this study. This finding might be associated with the skeletal structure of the 1st MTPJ. Specifically, the dorsal articular surface of the metatarsal is inclined towards the dorsal side of the foot and has a relatively wide surface area, allowing for the sliding of the base of the first metatarsal on its articular surface, thereby increasing the ROM of the 1st MTPJ extension (Fernández et al., 2016; Sichting et al., 2020; Zhang et al., 2022). The 1st MTPJ of FFS exhibited greater superior translation during the stance phase of 51%–82% than that of RFS, and the first metatarsal slid inferiorly relative to the first proximal phalanx during the extension process of the 1st MTPJ. This phenomenon may contribute to the larger extension angle of the 1st MTPJ during the 65%–75% of the stance phase in FFS compared with that in RFS. Accordingly, the present study found the 1st MTPJ extension angle was significantly greater (8.4°–10.1°) during the late stance (65%–75%) in FFS, consistent with the findings of the prior investigation. Bruening et al. (2018) found an increased MTPJ extension angle in FFS during the late stance compared with that in RFS. According to the windlass mechanism, as the 1st MTPJ extends, the plantar fascia is stretched, resulting in a decrease in the distance between the metatarsals and the calcaneus and an elevation of the medial longitudinal arch (Hicks, 1954), which is thought to further facilitating the transfer of the forces during late stance (Carlson et al., 2000). The greater extension angle of the FFS during push-off phase indicated that the plantar fascia experienced greater tension, resulting in increased arch height and stiffness and enhanced transmission of the propulsive effect (Goldmann and Brüggemann, 2012). By contrast, Chen et al. (2019) found that FFS resulted in greater compression of the medial longitudinal arch during mid and late stance phases, which led to a reduction of plantarflexion in the first metatarsal, thereby decreasing the dorsiflexion angle of the first phalanx. However, this controversy could be due to the fact that the study mentioned above only investigated the MTPJ motion of a single participant under barefoot conditions, while our study conducted an examination under shod conditions. Therefore, the greater superior translation and extension angle of the 1st MTPJ in FFS than in RFS during the late stance may enhance the propulsive effect.

Regardless of FSP, the 1st MTPJ consistently remained extension throughout the entire stance phase. This observation aligns with the findings of Bruening et al. (2018), who employed a marker-based infrared capture system to assess the sagittal plane motions of the 1st MTPJ during running at a velocity of 3.7 m/s. However, in their study, the minimal extension angles of the 1st MTPJ for FFS and RFS were confined between 5°–10°, with the maximal extension angles of the 1st MTPJ within 40°–45° for FFS and 35°–40° for RFS. These values were lower than the corresponding minimum (FFS: 17.4° ± 6.0°; RFS: 16.2° ± 7.3°) and maximum (FFS: 54.3° ± 7.0°; RFS: 50.7° ± 11.3°) extension angles of the 1st MTPJ in our investigation. Zhang et al. (2022) used DFIS and found the larger minimum and maximum angles of the 1st MTPJ compared to studies utilizing infrared motion capture systems during the stance phase of running. Therefore, previous research might underestimate the sagittal plane movements of the 1st MTPJ. The disparity in findings may be attributed to the differences in measurement instruments, specifically the use of marker-based motion capture systems versus the application of DFIS.

In the present study, the valgus angle of the 1st MTPJ between FFS (9.3° ± 10.6°) and RFS (7.5° ± 7.0°) was similar in the static CT model, and both angles were consistent with the clinical standard for the normal angle of the hallux (Menz and Munteanu, 2005). However, 4 out of 15 FFS runners exhibited greater than 20° of hallux valgus in the static CT model, whereas only one individual in RFS exhibited such a deviation, which corresponded to the clinical criteria for moderate hallux valgus (Iliou et al., 2016). In addition, we observed greater maximum valgus angle and ROM of varus to valgus in FFS compared with those in RFS. These findings might contribute to the higher incidence of moderate hallux valgus among FFS runners. Yu et al. (2020) used finite element simulation and discovered larger valgus angle of the 1st MTPJ results in increased stress exerted on the medial joint capsule. For runners with hallux valgus, running can contribute to the progression of this deformity (Heckman, 2000; Nagy, 2017). The larger maximum valgus angle in FFS during running may increase the risk of hallux valgus to some extent, especially for runners who already have symptoms of deformity.

This study has certain limitations. Firstly, the recruitment was limited to male runners. Secondly, the research was conducted under shod conditions, and the influence of footwear on the kinematic value of the 1st MTPJ was not investigated. Additionally, only one valid data set was selected for each trial for analysis. Future studies should consider these limitations and further examine the kinetics of the 1st MTPJ to gain a comprehensive understanding of its function during running.

## 5 Conclusion

DFIS was utilized to investigate the *in vivo* kinematic differences of the 1st MTPJ between habitual RFS and habitual FFS in shod running. The superior translation and extension angle of the 1st MTPJ were significantly greater in FFS than in RFS during the late stance, which may enhance the propulsive effect. However, the greater maximum valgus angle and the ROM of varus–valgus in FFS might potentially contribute to an increased risk of hallux valgus.

## Data Availability

The raw data supporting the conclusion of this article will be made available by the authors, without undue reservation.
